# Assembly of Dynamic P450-Mediated Metabolons—Order Versus Chaos

**DOI:** 10.1007/s40610-017-0053-y

**Published:** 2017-02-08

**Authors:** Jean-Etienne Bassard, Birger Lindberg Møller, Tomas Laursen

**Affiliations:** 1 Plant Biochemistry Laboratory, Center for Synthetic Biology, VILLUM Research Center “Plant Plasticity,” Department of Plant and Environmental Sciences, University of Copenhagen, Thorvaldsensvej 40, DK-1871 Frederiksberg C, Copenhagen Denmark; 2 Carlsberg Research Laboratory, Gamle Carlsberg Vej 10, DK-1799 Copenhagen V, Denmark; 3 Feedstocks Division, Joint BioEnergy Institute, Emeryville, CA 94608 USA

**Keywords:** Metabolon, Cytochromes P450, Channeling, Cytochrome P450 oxidoreductase, Membrane environment, Solvent environment

## Abstract

**Purpose of Review:**

We provide an overview of the current knowledge on cytochrome P450-mediated metabolism organized as metabolons and factors that facilitate their stabilization. Essential parameters will be discussed including those that are commonly disregarded using the dhurrin metabolon from *Sorghum bicolor* as a case study.

**Recent Findings:**

Sessile plants control their metabolism to prioritize their resources between growth and development, or defense. This requires fine-tuned complex dynamic regulation of the metabolic networks involved. Within the recent years, numerous studies point to the formation of dynamic metabolons playing a major role in controlling the metabolic fluxes within such networks.

**Summary:**

We propose that P450s and their partners interact and associate dynamically with POR, which acts as a charging station possibly in concert with Cyt*b5*. Solvent environment, lipid composition, and non-catalytic proteins guide metabolon formation and thereby activity, which have important implications for synthetic biology approaches aiming to produce high-value specialized metabolites in heterologous hosts.

## Introduction

**Fig. 1:**
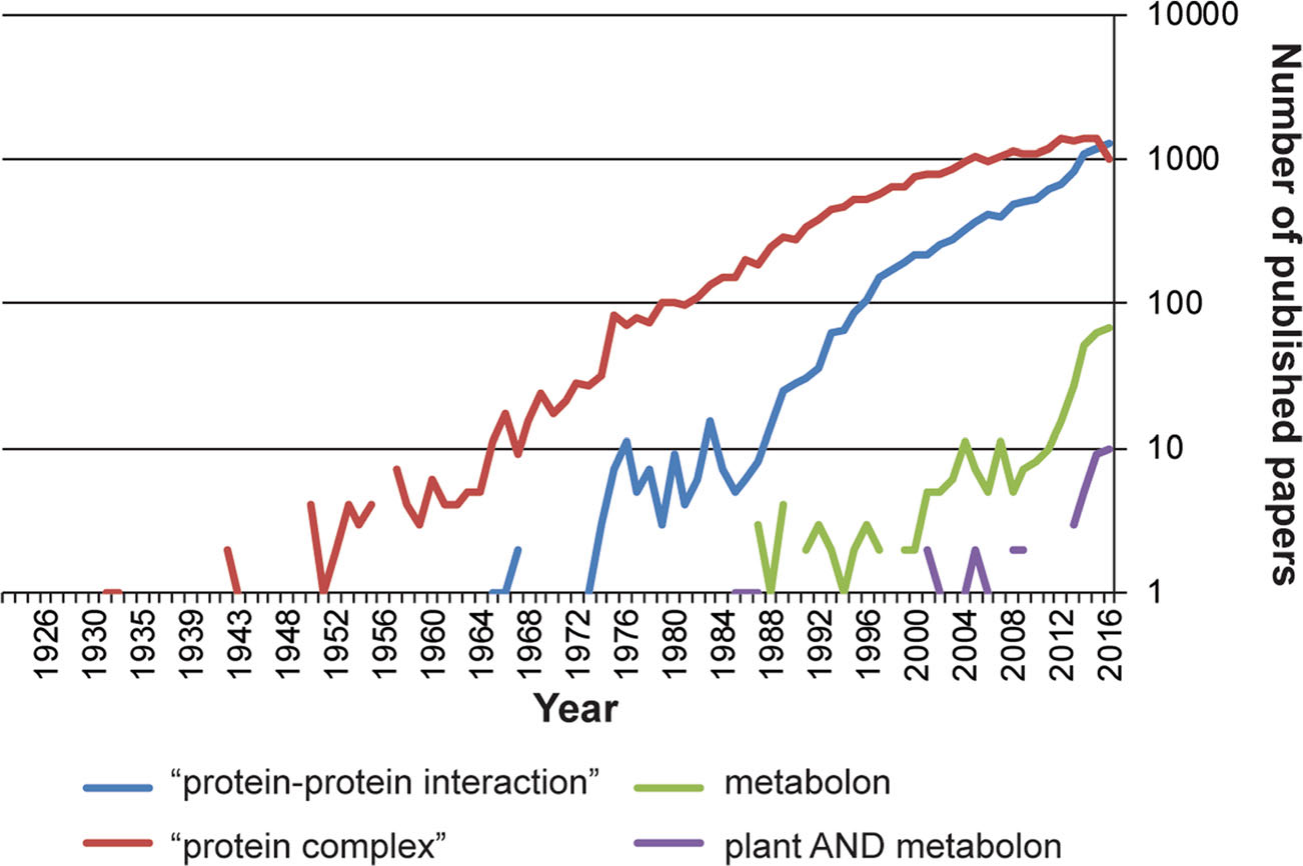
Pubmed occurrences about metabolon papers. Pubmed (https://www.ncbi.nlm.nih.gov/pubmed) queried towards the end of November 2016. With the queries: “protein-protein interaction,” “protein complex,” metabolon, and plant and metabolon

As sessile organisms, plants produce an impressive diversity of toxic chemical compounds to defend themselves from the continuum of biotic and abiotic challenges they are exposed to. The specialized metabolites produced depend on the specific plant species and type of challenges encountered, and their biosynthesis demands a high degree of functional organization to offer swift metabolic plasticity. Delicate mechanisms regulate the complex metabolic grids organizing biosynthesis and storage [1••]. Organellar compartmentation allows cells to bring enzymes and their substrates in close proximity, which serves to increase local concentration for biological process optimization. In plants, many pathways involved in both general and specialized metabolism have been proposed to organize in protein complexes, metabolons [2•]. This extends the idea of compartmentalization at the molecular level. Rapid metabolic reconfigurations, as required for biosynthetic diversity, may be controlled by formation of dynamic metabolons [3] facilitating channeling of labile and toxic intermediates out of the bulk phase, increasing local substrate concentrations and preventing undesired metabolic cross talk [4]. Dynamic assembly and disassembly permit swift adaptation of the metabolite profile to environmental challenges [5, 6]. Beyond the fundamental understanding of cellular metabolism, it has become increasingly important to understand how enzyme systems catalyzing complex reactions are spatially organized and their possible enrolment as part of dynamic metabolons. Intensive efforts to maximize product yield from genetically engineered pathways were undertaken without much attention to this aspect [7–9]. Current synthetic biology approaches based on the function of biological modules and the possibility to combine these in new ways are challenged up front by constraints arising from lack of understanding the impact of proper structural organization [10, 11•, 12]. The concept of metabolon formation in plants and animals was first suggested in 1974 [13] and predicted to be of relevance to both general and specialized metabolism. Pathways known to involve the formation of metabolons include the dhurrin pathway [14, 15••], the core phenylpropanoid pathway [5, 16], the flavonoid pathways [17, 18], the isoflavonoid pathways [19•, 20], the biosynthesis of sporopollenin subunits [21], the TCA cycle [22•], the Krebs cycle [23•], the Calvin-Benson cycle [24], the biosynthesis of bitter acids in hop [25], the biosynthesis of lipids [26], the fatty acid biosynthetic network [27], the spermine/spermidine synthesis [28], the photosynthetic complex [29], the auxin synthesis [30, 31••, 32], the cholesterol synthesis [33], the de novo purine synthesis [34•], the urea cycle of rat hepatocytes [35], the citrate cycle of porcine liver [36], the glycolytic pathway in rabbit skeletal muscle and mouse fibroblasts [37, 38] and the glycolytic pathway in *Saccharomyces cerevisiae* [39]. Interested readers can read some of the excellent reviews written over the years on this topic [1••, 2•, 13, 14, 40–42]. Despite this rather exhaustive accumulation of data, there is still skepticism about the necessity of metabolons for organizing cellular metabolism mainly due to the technical challenges encountered in efforts directed towards isolating the active complexes. However, scientific papers on this subject have accumulated and the number of papers mentioning metabolons has rapidly increased within the last 5 years (Fig. 1).

In this review, we will focus mostly on recent papers and use the dhurrin metabolon as a case study. Dhurrin is a cyanogenic glucoside present in the cereal crop plant *Sorghum bicolor*. Dhurrin is produced from the amino acid l-tyrosine and synthesized by two membrane-anchored cytochrome P450 enzymes (CYP79A1 and CYP71E1) and by a soluble UDP-glucosyltransferase (UGT85B1). Although multiple studies have focused on the formation of metabolons involving P450 enzymes, and even if metabolon formation seems essential to organize and direct flux across biosynthetic networks [6], it is still unknown how the dynamic assembly of such complexes is regulated and organized. Here, we will illustrate different possible models of organization, which are best in agreement with the published data. In the first parts of this review, we will discuss briefly factors that are classically considered when studying metabolon formation, i.e., channeling of intermediates and protein-protein interactions. Then, we will review more extensively factors that are likely to regulate the dynamic assembly process, i.e., membrane environment, solvent environment, non-catalytic proteins and higher-order clustering, that are still largely disregarded.

## Definition and Challenges of P450-Based Metabolons

The expansion of the plant-specialized - metabolites during evolution notably results from the massive recruitment of cytochromes P450 (P450s) that catalyze multiple types of catalytic reactions [43]. Eukaryotic P450s are type-II membrane-anchored enzymes and proposed as nucleation platforms anchoring metabolons to the endoplasmic reticulum (ER), i.e., in the lignin [5], the isoflavonoid [19•], and the dhurrin biosynthetic pathways [15••]. The plant ER is a dynamic multitasking organelle connected to several compartments of the cell and involved in cell processes like protein synthesis, signaling, and primary and secondary metabolism [44]. The ER membrane is highly crowded with proteins [45], which points to a tight organization of the ER proteins to avoid hazardous crosstalk and to prevent inadvertent competition for common substrates between overlapping P450-mediated metabolic pathways.

### Channeling, Protein-Protein Interactions and Allosteric Regulation

Metabolons are defined as organization of sequential biosynthetic enzymes into complexes and involve substrate channeling and protein-protein interactions. Furthermore, metabolons involved in specialized metabolism are commonly considered highly dynamic, responding to environmental conditions. Initially, substrate channeling studies pioneered the investigation of metabolons [46], providing evidence for intermediate channeling between two consecutive enzymes of the phenylpropanoid pathway, the phenylalanine ammonia lyase (PAL) and the cinnamate 4-hydroxylase (C4H), a P450. Although the results of succeeding studies on channeling between these enzymes have been contradictory [47, 48], this phenomenon has been observed in several pathways and remains a key feature of metabolons [2•, 14, 41]. Another key parameter is the occurrence of multiple weak protein-protein interactions leading to stabilization of larger complexes. More and more studies demonstrate protein interactions in virtually all parts of plant metabolism, including the phenylpropanoid pathway [5, 19•, 21, 49], the dhurrin pathway [15••, 50], and lipid biosynthesis [26]. Complex formation also involves P450-P450 complexes [51••] with reported oligomers ranging from dimers up to 40-mers [52]. In eukaryotes, P450s are nearly always present as membrane proteins, with N-terminal hydrophobic amino acids forming an α-helix anchor to the ER membrane and their catalytic domain facing the cytosol. In this manner, P450s constitute a nucleation platform facilitating localization of metabolons to the ER. Evidence has shown that metabolism of substrates by a given P450 may be influenced by the specific interaction with other P450s [51••], pointing to competition or allosteric effects between P450s. Thus, the last parameter typically considered is the allosteric effect between enzymes involved in metabolon formation. For example, we observed that the mere physical presence of UGT85B1, even in the absence of its UDP-glucose cofactor, stimulated the catalytic properties of CYP79A1, a non-direct partner in the dhurrin pathway [15••], supporting the assembly of a metabolon in planta.

### Stoichiometric Imbalance Leads to Dynamic Assembly

The catalytic cycle of all microsomal P450s requires two single-electron transfer reactions catalyzed by the shared NADPH-dependent cytochrome P450 oxidoreductase (POR). This makes POR an essential component of P450-mediated metabolism, even if it is typically not considered in the understanding of metabolic networks and metabolons. POR, as P450s, is an ER-anchored protein, and the physical interactions between P450s and POR are facilitated and modulated by the membrane environment [53, 54]. It is still not clear if electron transfer from POR to a P450 enzyme requires formation of a 1:1 functional complex [55] or the involvement of higher-order multimers of the P450s [51••, 56]. In most organisms, the P450s are present in a large excess in comparison to POR with reported stoichiometric ratios from 5:1 to 40:1 [15••, 52, 57–61]. Nevertheless, POR must provide electrons to each of the different P450s present. This may be controlled by the multiple functional states [62] and conformational dynamics of POR [63, 64]. The large number of different P450s compared to the presence of sub-stoichiometric amounts of POR has prompted the hypothesis of transient interactions between P450s and POR. P450-mediated metabolism may therefore be limited by the POR availability. Thus, highly complex and efficient regulations must occur to ensure the coordinated operation of all P450s belonging to various biosynthetic pathways. P450-P450 interactions can alter P450-mediated metabolism by affecting their ability to interact with POR, to bind substrate, or by both types of mechanisms [51••]. In contrast to yeasts and mammals, the P450-POR couple is even more complex in plants. Plant POR isoforms usually group into two phylogenetically distinct classes [65, 66]. The number of POR isoforms present within a single plant species is between one and four, and they may be derived from a single or both phylogenetic classes [67]. The two POR classes have not been shown to display structural specificity towards individual P450. Direct in vitro interaction and reduction assays of P450s demonstrated that both POR classes performed equally well [66]. However, differential expression patterns of the two classes suggest functional distinct roles. The POR class I enzymes are constitutively expressed and suggested to mainly be involved in primary and basal specialized metabolism. The class II PORs have an adaptive role in specialized metabolism [65, 66] responding to environmental stimuli such as wounding, pathogen infection, or light exposure [67–70]. The hypothesis is that class II POR provides electrons for highly expressed P450s involved in “on-demand” specialized metabolism. This highlights the evolutionary strategy that ensures an efficient activation of P450s in planta.

## Microenvironments Regulate Metabolon Assembly: an Unknown Terrain

Accumulating experimental data support the importance of individual protein-protein interactions in order to accomplish substrate channeling. However, the experimental approaches used typically involve targeted screening based on prior knowledge. Very little is known about the triggering mechanisms that stimulate assembly of metabolons and the biomembrane microenvironments required to stabilize these dynamic complexes. Here, we will highlight three areas that remain poorly understood: the neighboring proteins not essential for in vitro functional studies, the lipid bilayer environment, and the local solvent medium properties.

### Neighbor Proteins in the Microenvironment

In animals and yeast, a small number of proteins have been found associated to P450s without being directly involved in their catalytic function. BiP proteins are co-detected with P450s and seem important for their ER retention [71]. CYP2C2 has been shown to interact with the ubiquitous BAP31 integral membrane protein [72] and reported to associate with the cytoskeletal components actin and myosin [73]. Erg28p has been reported to interact with CYP51 in yeast. The Erg28p protein was proposed to tether a large complex of yeast sterol biosynthetic enzymes [74]. In plants, several ER-resident non-catalytic protein partners have repeatedly been found associated together: P450s, POR, cytochrome *b5* (Cyt*b5*), reticulons, synaptotagmin (SYTA), band7 protein, sterol methyltransferase (SMT), vesicle-associated protein (VAP), membrane steroid-binding protein (MSBP) and others [5, 15••, 19•, 75]. These proteins are typically not required for activity in vitro and are generally ignored, but seem to be part of the microenvironment around metabolons and might have a role in their stabilization and activity.

Hildebrandt and Estabrook first reported the involvement of Cyt*b5* in P450-catalyzed reactions [76]. Cyt*b5* is a small (15 kDa) hemoprotein known to enhance the activity of several P450s, thus increasing the complexity of the electron donation chain of P450-mediated metabolism, but still largely overlooked. While Cyt*b5* is usually considered as non-essential for P450 activity in vitro, several studies have demonstrated the complex in vivo functional role of Cyt*b5* in P450 catalysis [77]. POR is required for delivering the primary single electron to P450s, whereas both POR and Cyt*b5* can provide the electron for the second electron-transfer step. Cyt*b5* has been shown to stimulate, inhibit, or have no effect on various P450 reactions [77, 78]. Despite this seemingly elusive functional importance, Cyt*b5* has a significant role in forming hetero-dimers with P450s either to provide electrons or induce conformation changes augmenting interaction between POR and selected P450s [78]. NADH:cytochrome *b5* reductase and Cyt*b5* may even serve as sole electron donors to the human CYP1A1 [79]. Most of the studies have focused on mammalian Cyt*b5* proteins. However, several isoforms of Cyt*b5* are also found in plants and most likely display similar roles [80]. Various approaches pointed to Cyt*b5* importance for P450 function in plant phenolic metabolism [81] and in fatty acid hydroxylations [82–84]. Cyt*b5* is not essential in glucosinolate biosynthesis but can modulate P450 activities and change the glucosinolate pattern [80]. Cyt*b5* isoforms have been found associated to the lignin metabolon [5] and the dhurrin metabolon [15••]. *Arabidopsis* *thaliana* Cyt*b5* proteins interact with a series of other proteins in addition to the P450s, such as a plasma membrane-localized sucrose transporter [85] and the ethylene-response regulator RTE [86]. This suggests that Cyt*b5* could play a broader important physiological role. Understanding the role of the Cyt*b5* isoforms may have huge implications for synthetic biology approaches to boost P450 metabolism [87••].

Reticulons contribute to ER tubule shaping [75, 88]. Vesicle-associated membrane protein (VAMP)-associated proteins (VAPs) are localized at the ER-plasma membrane contact sites (MCS), and VAPs serve to properly associate ER, cytoskeleton, plasma membrane and cell wall [89]. Plant SYTA is reported to be plasma membrane protein [75, 90], while other synaptotagmins in animal and yeast cells (called tricalbins in yeast) are ER-localized. Synaptotagmins form ER-plasma membrane junctions maintain ER morphology, stabilize the MCS and are involved in intracellular lipid trafficking, Ca^2+^ signaling and exo/endocytosis [89, 91]. Reticulons and VAPs effect endosome contact, endocytosis and even auxin homeostasis [92]. The band7 protein is a homolog of the ER lipid raft-associated proteins (erlins) found associated with high molecular mass protein complexes in mammalian cells [93, 94]. Accordingly, several of the proteins repeatedly found to be associated with P450s and metabolons are involved in ER membrane homeostasis and sterol and lipid homeostasis. This supports the hypothesis of the requirement of a specific environment for the establishment of ER membrane-anchored metabolons.

### Membrane Environment: a Dynamic Regulatory Scaffold

Several biosynthetic pathways are distributed across different organelles and even different cells or have products excreted by cells [95–100]. MCS in animals have been shown to be involved in lipid trafficking and in other functions such as organelle dynamics, protein import and primary metabolism [101]. The metabolon formation theory is compatible to this organization of metabolic pathways. Subparts of biosynthetic pathways might be organized in different metabolons across different organelles with transporters and neighboring proteins in the metabolon environment making bridges between the different biosynthetic locations. The plant endoplasmic reticulum is an organelle connected to several compartments of the cell, cytoskeleton [102, 103], Golgi [104], chloroplast [105], plasma membrane [102, 106], peroxisomes and mitochondria [107, 108]. Physical contact sites, named plastid-associated membranes (PLAMs), have been demonstrated between the chloroplast and ER membranes [109]. PLAMs have specific lipid compositions which differ from chloroplast and ER [101]. Hemi-fused membranes at PLAMs facilitate inter-organellar interactions and allow enzymes on both membranes to process non-polar compounds from both organelle membranes in a transporter-independent manner [105, 110••]. This would allow deployment of metabolic intermediates from enzymes/metabolons directly into the leaflet of the adjunct organelle. Some metabolons could be settled in or around MCS and PLAMs. Proteins repeatedly observed to co-purify with P450s could be part of these structures.

The ER obviously functions as a highway for delivery of a diverse range of proteins and molecules [31••]. Quantified by in planta fluorescence correlation spectroscopy [15••] or fluorescence recovery after photobleaching [5], P450s and partner soluble enzymes are highly mobile at the ER membrane surface or in the cytoplasm. P450s and therefore also P450-containing metabolons associated to the ER are moving together with the dynamic and constantly remodeled ER structures [46]. Microenvironments containing enzymatic production sites, metabolons, for defense molecules could benefit from targeted actin-guided transport through ER dynamics in response to pathogen attack or for localization near pathogen entry sites to facilitate production of high local concentrations of defense compounds [5, 6, 14, 15••, 111]. Indeed, increase in ER structures on exposure to stress such as wounding has been suggested to have a role in defense against pests and pathogens [112–116]. Metabolons involved in production of cell polymer subunits may also depend on the ER dynamics, as ER structures increase during pollen tube growth [44].

Eukaryotic P450s and POR are both membrane anchored and thought to partly interact via their transmembrane N-amino acid terminus [51••, 71]. Local changes in lipid environment are therefore envisioned to play a key role in controlling and stabilizing metabolon assembly. The increasing understanding of the membrane during the last decade has indeed revealed that the membrane bilayer does not simply act as a passive 2D lattice but actively influences various cellular processes such as transport, protein trafficking and signaling. Independent sets of data show that the catalytic properties of P450s are also modulated by membrane composition [15••, 51••, 117•]. Lipids with varying acyl chain length and head groups may associate to generate lipid domains with different electrostatic surface charges. Lipid membranes can adopt two distinct phases referred to as non-fluid liquid-ordered domains and fluid liquid-disordered domains. The existence of lipid microdomains may direct the selective clustering of proteins into the same specific regions of the membrane increasing their propensity to interact. Modulation of membrane lipid composition under varying environmental conditions is an important part of plant adaptation [118], which would therefore also be expected to impact metabolon establishment. Liquid-ordered domains have been implicated in numerous cellular processes and could act as platforms for metabolon formation [119]. The catalytic properties of animal P450s and POR have also been found to be regulated by their presence in disordered or ordered domains [120]. Some lipid-mediated effects result from direct interactions of P450s and POR with phospholipids. These interactions can affect P450 function by altering their conformation, by affecting the interactions between P450s and their redox partners, or by altering the redox potential of the electron donor POR [51••, 117•, 121, 122]. Animal studies have suggested that P450s are enclosed in a “phospholipid halo”, possibly affecting the diffusion of substrates and products [123]. The specific membrane composition of liquid-ordered membranes has been shown to increase binding efficiency between CYP1A2 and POR [117•, 120]. In the absence of POR, P450s have been shown to possess a greater affinity for negatively charged phospholipids [117•]. CYP2B1 was shown to modulate membrane properties in reconstituted systems by inducing segregation of anionic phospholipids, which resulted in alterations in the activity and conformation of CYP2B1 [124]. The dhurrin P450, CYP71E1, was particularly affected by lipid charges displaying maximal activity in membranes containing 20–30% negatively charged phospholipids [15••]. These findings point to the important but still underestimated fact that plant ER membranes are heterogeneous and that the membrane itself is an integral part of the metabolon [6]. Lipid composition might just be the tip of the iceberg, and several other components of the microenvironment could have a role in metabolon formation and activity.

### Controlling the Local Media Based on Intracellular Heterogeneity

Cells have generally been thought of as composed of two solvent environments, namely the hydrophilic aqueous phase and the hydrophobic lipid phase. Increasing evidence suggests that this is a too simplified view and that the cellular cytosol is a marbled soup of solvent microenvironments. This implies that local concentrations of solutes, salt and pH may differ and control metabolism in a hitherto unknown fashion [125••]. Intracellular heterogeneity may proceed from demixing of macromolecules and small molecules as exemplified by the formation of P granules of RNA and protein [126]. A few general metabolites including sugars, some amino acids, choline, and some organic acids such as citric acid, tartaric acid, succinic acid, lactic acid and malic acid are always present in considerable amounts in all microbial, mammalian and plant cells and cannot be only considered as intermediates in metabolic pathways [127•]. When mixed in precise ratios and in the absence of water, these simple molecules become viscous liquids, termed natural deep eutectic solvents (NADES) [128]. Thus, NADES have been proposed as a third cell phase [129]. NADES provide 50–100 times increased solubility of many poorly soluble specialized metabolites and may therefore enable solubilization and storage of various toxic and unstable metabolites in cells [127•, 128, 129] (Knudsen et al., submitted). The occurrence of NADES in the plant cell may explain that compounds like flavonoids and anthocyanins occur at levels above their solubility in both water and lipids [129]. NADES may provide stability towards several stresses such as light, temperature and time [130]. We have recently discovered that NADES provide an excellent inert environment for storing enzymes, which upon dilution recover their activity and pinpoint how plants may tolerate desiccation (Knudsen et al., submitted).

ER components might be associated with NADES, e.g., with the positively charged choline head groups in the ER membrane associating with sugars and acids and contributing to the formation of a NADES. P450 metabolons might be dissolved in this solvent together with substrates and intermediates. Thus, NADES could make a kind of shield around metabolons and create a microenvironment within the cytoplasm, making experimental studies to isolate the metabolon even more complex [5, 129]. NADES acting as a shield may explain the close interaction between enzymes in dynamic metabolons without the need for strong interactions.

## Organization of Plant P450 Metabolons: a Case Study of the Dhurrin Metabolon

The plant ER is a multitasking membrane network connected to several organelles and involved in most cellular processes [44]. Consequently, P450s and related metabolons are in close vicinity to several other enzymes and non-catalytic proteins, all of which must be well organized. The precise organization and stoichiometry of individual components present remain poorly understood. Here, we propose several scenarios based on previously published data [15••] and considering the factors most likely to modulate metabolon assembly and activity (see above). The plausibility of each of these scenarios is evaluated pointing to the model 5 presented in Fig. 4b.

**Fig. 2:**
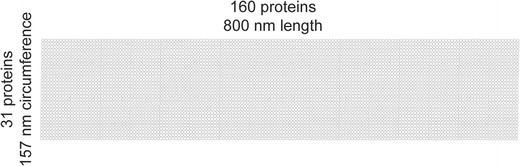
Representation of a protein array at the ER surface. ER-anchored protein molecules are each represented by a *white* *dot*. A total of 4960 protein molecules are displayed

The ER membrane system can form diverse structures, e.g., sheets, cisternae, and tubules, but most commonly is tubular with a reported average ER tubule diameter of 50 nm [131–134]. With an ER tubule circumference of about 157 nm, an annulus at the circumference of an ER tubule membrane is envisioned to consist of an average of 31 proteins each with an averaged diameter of 5 nm. All cellular membranes including the ER are considered highly crowded by proteins [45]. Accordingly, we schematize the ER membrane proteins as a layer of contiguous proteins delimited by the circumference and the length of the ER structure where they reside. An illustration of 4 pixels in confocal images and movies [15••] of ER tubules would measure approximately 800 nm in length and 50 nm in diameter and contain an average of 4960 proteins (Fig. 2).

For clarity of the further models, we do not display UGT85B1 and other soluble proteins or scaffold proteins that might mediate metabolon formation as discussed earlier.

### Model Requirements and Constraints

In order to propose a model describing the current experimental data on the dhurrin metabolon, we have defined seven constraints essential for the validity of the model. We explore five models and validate them according to the constraints, where green “√” indicates that the constraint is fulfilled, the orange “(√)” indicates that the constraint may be satisfied in very specific conditions, and the red “X” indicates that the constraint cannot be explained by the configuration proposed.

Constraint #1: Based on quantitative proteomics data [15••], we calculated that for a total number of 100,000 ER protein molecules present on the ER, the number of protein molecules representing the dhurrin biosynthetic enzymes is as follows: 75 ± 3 POR2b, 238 ± 37 CYP79A1, 446 ± 181 CYP71E1 and 516 ± 95 other P450s. From *S. bicolor* total extract data, we calculated an abundance of 134 ± 2 UGT85B1 molecules within a total number of 100,000 proteins. Accordingly, we define constraint #1: ER membrane surfaces harboring a total number of 4960 proteins must display 4 ± 0 POR2b, 12 ± 2 CYP79A1, 22 ± 9 CYP71E1, 26 ± 5 other P450s and 4896 other ER proteins. P450s are in large excess compared to POR, whereas the UGT85B1 abundance is similar to that of CYP71E1. More than half of the total P450s are CYP79A1 and CYP71E1, as previously determined by spectroscopy [135]. Most importantly, all the dhurrin proteins are highly diluted in their native ER environment and even to a higher degree in context of total cellular protein. Even though CYP79A1, CYP71E1 and POR2b represented less than 0.8% of the total protein content of the ER membrane, they are able to interact specifically and form a dynamic metabolon.

Constraint #2: Using the Förster resonance energy transfer (FRET) equation with parameters of the eGFP-mRFP1 pair [15••], we have the relation between fluorophore distances and FRET values. FRET is calculated using the following equation:

$$ \mathrm{FRET}=\left(\frac{R_0^6}{R_0^6+ R}\right) $$

with *R*_0_ representing the Förster radius and *R* the distance between donor (eGFP) and acceptor (mRFP1). For the eGFP-mRFP1 couple, *R*_0_ is equal to 4.7 nm [136]. Therefore, a measured FRET of 20% corresponds to an average distance between fluorescent proteins (center to center) of 5.9 nm, 5% FRET represents an average distance of 7.7 nm, 0.1% FRET represents an average distance of 14.9 nm, and finally, no FRET measured reflects distances above 15 nm. Considering proteins of an average diameter of 5 nm (average P450 diameter), we can record FRET between two proteins if no more than one protein is between the two targets. Above 5% FRET, no protein could interspace the target proteins. Thus, constraint #2a is as follows: High FRET values are measured only when proteins are interacting, forming oligomers (dimers or higher oligomers). In certain conditions, co-expression of a third partner protein enhanced the FRET values between the two other partners, e.g., increased FRET was observed between UGT85B1 and CYP79A1 fusion proteins upon co-expression of untagged CYP71E1 [15••]. This “additive” effect is constraint #2b. Increase in FRET average is the consequence of an increased spatial proximity of the two studied fluorescent proteins or an average of longer or more frequent interactions.

Constraint #3: Background FRET values measured between two unrelated ER proteins ranging from 0 to 5% [15••] define constraint #3. Non-interacting proteins display FRET values lower than 5%, when residing in the same micro-domain or meeting stochastically in a restricted volume as the ER membrane.

Constraint #4: When a protein A interacts with a protein B and concurrently protein B interacts with protein C, proteins A and C may be envisioned to be drawn very close to each other resulting in indirect FRET. High indirect FRET values were measured between non-partners at some very specific conditions [15••], and this phenomenon represents constraint #4.

Constraint #5: At the resolution of the confocal microscope, a uniform fluorescence emission was observed from the ER membrane system expressing the fluorescent-labeled dhurrin pathway enzymes [15••], although brighter areas indicated higher local concentrations of the tagged proteins. Hence, the dhurrin metabolon assembles in multiple clusters along the ER network. Thus, constraint #5 is as follows: the continuous fluorescence detected across the ER network, including local brighter areas.

Constraint #6: FCS experiments highlighted high dynamics of the target proteins, high variability of diffusion speeds, and decreased speeds of each target protein when partner proteins were co-expressed [15••], which defines constraint #6.

Constraint #7: Metabolomic data demonstrated efficient channeling upon metabolon formation without leakage of intermediates or by-products [15••] and describes constraint #7.

**Fig. 3:**
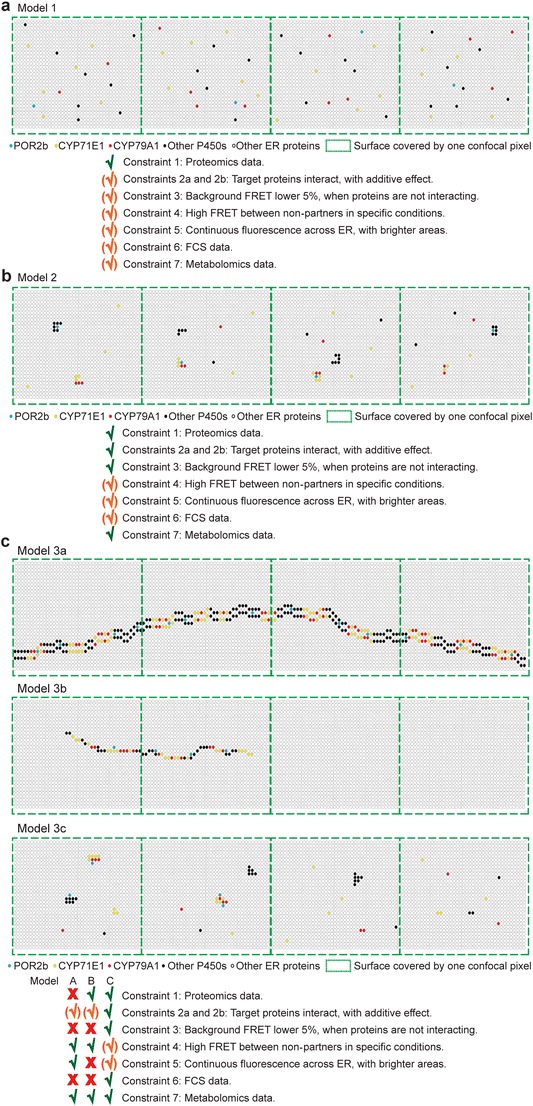
Models 1, 2, and 3. **a** Model 1 of a random distribution of dynamic proteins. All the proteins are freely moving and only interact by random “hit-and-run” mechanism. **b** Model 2 of stable metabolons. This model involves pre-formed metabolons that are exchanged to the POR acting as a charging station. **c** Models 3a, b and c of proteins organized in large structures. Model 3a, proteins organized in highways across the ER tubule with POR trapped inside the highways. Model 3b, proteins organized in highways across the ER tubule with POR freely moving around the highways. Model 3c, proteins organized in aggregates with freely moving POR

Model 1 (Fig. 3a) assumes a random distribution of all proteins and only fully meets the criteria of constraint #1. Part of other constraints could be fulfilled if proteins are highly dynamic and partners display strong affinity for each other. These two conditions increase the probability of protein-protein interactions without release of intermediates. Nevertheless, protein associations driven by strong protein-protein interactions will inevitably lead to formation of classical stable enzyme complexes and high-order aggregates. The entire dhurrin metabolon has never been purified in its ensemble, which points to transient and weak protein-protein interactions [15••]. Finally, formation of large aggregates would result in observable ER membrane surface areas depleted in target proteins, which disagrees with the continuous fluorescence observed. *Consequently, this model was discarded.*

Model 2 (Fig. 3b) represents an exchange of metabolons close to POR, which behaves as a hub for providing electrons for specific P450s. P450s not interacting with POR are not functional. To fulfill constraint #6, not all proteins of the same type can be permanently forming a metabolon, requiring non-associated proteins and thus an exchange between free and bound proteins. Constraint #4 requires functionally distinct metabolons in close proximity or large mixed complexes. However, such organization would inevitably lead to depletion of fluorescence or high heterogeneity in confocal images. Such large complexes are not in compliance with the required dynamic nature. Again, the dhurrin metabolon has never been purified in its ensemble, which points to weak protein-protein interactions. *Consequently, this model was discarded.*

Models 3a, b, and c display three different configurations of P450s organized in highways of large clusters (Fig. 3c). The constraints #1 and #5 cannot be satisfied at the same time if studied proteins are organized in large structures. With these kinds of large complexes composed of mixed metabolons, high FRET values would be recorded from unrelated proteins. To fulfill the constraint #6, some of the proteins studied have to exist in a free, non-metabolon-associated state. As explained previously, the dhurrin metabolon has never been purified in its ensemble, pointing to the formation of non-stable complexes. *Consequently, this model was discarded.*

**Fig. 4:**
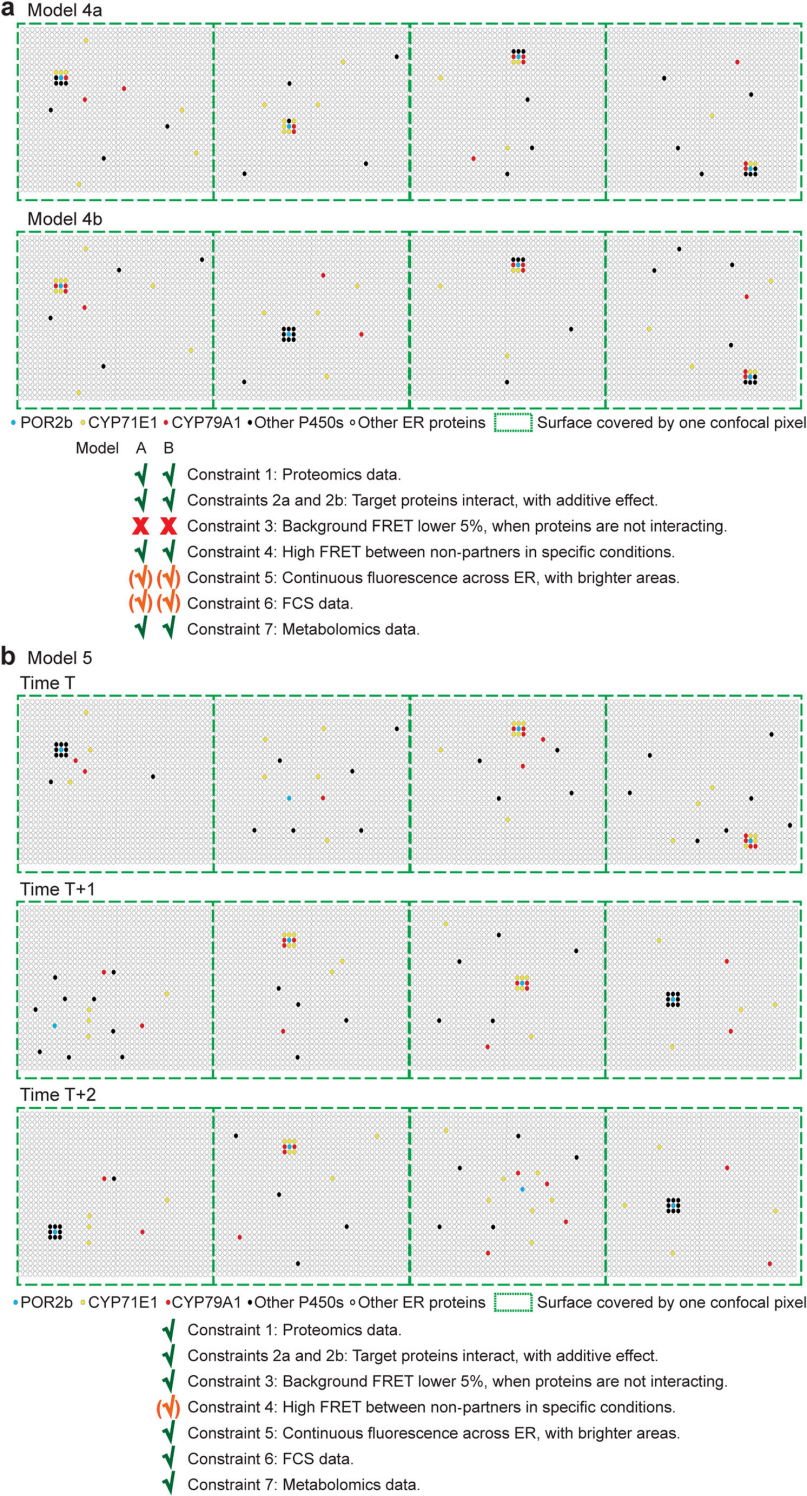
Models 4 and 5 of proteins organized as dynamic metabolons. **a** Model 4a, mixed metabolons are formed around the POR acting as a charging dock. Model 4b, POR encircled by one or mixed metabolons. **b** Model 5 of formation of specific transient and dynamic metabolon around the POR. Only one metabolon is present at a time *t* around the POR. This model implies a dynamic exchange of metabolon components around a POR acting as a charging station. Metabolons could be formed by oligomers of each component. Association and dissociation of metabolons are controlled upon cellular needs. Three arbitrary time points are shown in this figure

Model 4 displays two models (Fig. 4a) revisiting the earlier model of P450s encircling PORs [137]. These models do not meet three of the experimental constraints. Firstly, if P450s involved in competitive metabolons are permanently associated around the same POR, this will result in high FRET between unrelated P450s, which was not detected in Laursen et al. [15••]. Secondly, free, non-associated proteins are required to satisfy constraint #6. Thus, continuous exchange of P450s around the PORs is expected. Thirdly, a repetitive similar protein organization across the ER network is a requisite for the observed continuous fluorescence. *Consequently, we discard these models.*

Model 5 proposes that specific P450s organize in dynamic metabolons around POR (Fig. 4b). To satisfy constraint #4, a continuous exchange of P450s around PORs is required, provoking association and dissociation of competitive metabolons. The exchange of P450 interactions between non-partners will, as observed, result in increased background FRET [15••]. Furthermore, restriction of PORs and P450s to micro-domains in the membrane will increase the probability of FRET between non-partners at the surface of ER. All the other constraints were satisfied; thus, *we favor this model.* This model satisfies observations of homo- and hetero-oligomerization of CYP79A1, CYP71E1 and UGT85B1: that CYP79A1 and CYP71E1 recruit UGT85B1, and that UGT85B1 interacts with both CYP79A1 and CYP71E1 but is not necessary for CYP79A1-CYP71E1 complex formation. UGT85B1 is situated close to the non-partner ER membrane proteins, CYP98A1 and POR2b, during association and dissociation of the metabolon, when CYP79A1 and CYP71E1 are co-expressed.

The model proposed here suggests that P450s and soluble partner enzymes interact with POR, which acts as a charging station possibly in concert with Cyt*b5*. Lipid composition and non-catalytic proteins might guide metabolon formation and control P450 activity as discussed above. The existence of NADES might also limit leakage of metabolic intermediates by isolating the metabolon components from the cytosol.

## Conclusion

On-demand formation of specialized metabolites enables plants to respond to environmental stresses through the sequential action of multiple enzymes. Fine-tuning of the metabolism is accomplished through dynamic metabolons to provide the necessary plasticity for plants to survive the continuum of biotic and abiotic challenges. The question remains, how such dynamic metabolons are organized, despite the facts that (1) P450 metabolons are localized at the dynamic ER surface, (2) metabolon components are highly diluted in plant cells, (3) the ER is highly crowded, and (4) the ER is a multifunctional and dynamic organelle site of protein production, lipid biosynthesis [138], auxin regulation [139], calcium homeostasis [140], and oil and protein body formation [141]. In this review, we argue that all proteins involved in metabolon formation must be tightly regulated and organized facilitating substrate channeling without release of metabolic intermediates. Our proposed model (model 5, Fig. 4b) integrates the current knowledge of the dhurrin metabolon. We propose that P450s interact and associate with POR to form transient platforms for recruitment of soluble partner enzymes. The lifetime of metabolons still remains largely unknown mainly due to technological limitations. A metabolon with changing composition and/or numbers of proteins has the potential to provide additional regulatory power under changing environmental and developmental situations.

Cellular metabolism is highly modular utilizing relatively few enzyme classes to generate an impressive diversity of metabolites. Therefore, we hypothesize that other biosynthetic pathways, especially those involved in formation of specialized metabolites, may be organized in a similar fashion as the dhurrin metabolon. Understanding the mechanisms that govern metabolon assembly has huge implications for synthetic biology approaches aiming to produce high-value plant specialized metabolites in heterologous hosts, which often suffer from low yield and release of toxic intermediates [6, 15••, 142, 143••]. Optimizing yield and titers may require co-expression of additional non-catalytic proteins, changing the lipid environment and intracellular metabolite profile.
